# Water *versus* Asphaltenes; Liquid–Liquid and Solid–Liquid Molecular Interactions Unravel the Mechanisms behind an Improved Oil Recovery Methodology

**DOI:** 10.1038/s41598-019-47782-5

**Published:** 2019-08-06

**Authors:** Edris Joonaki, Jim Buckman, Rod Burgass, Bahman Tohidi

**Affiliations:** 1 Centre for Flow Assurance Research Studies (CFAR), Institute of GeoEnergy Engineering, School of Energy, Geoscience, Infrastructure and Society, Heriot-Watt University, Riccarton, Edinburgh EH14 4AS UK; 2 Centre for Environmental Scanning Electron Microscope, Institute of GeoEnergy Engineering, School of Energy, Geoscience, Infrastructure and Society, Heriot-Watt University, Riccarton, Edinburgh EH14 4AS UK

**Keywords:** Crude oil, Petrol

## Abstract

Understanding of possible molecular interactions at liquid-liquid and solid-liquid interfaces can shed lights onto the nature’s design and authorise fine manipulation aptitude in biological, manufacturing, microfluidic and oil recovery applications. Of particular interest is the capability to control the aggregation of organic and biological macromolecules, which typically poses significant challenges for oil industry and human life, respectively. Following asphaltene aggregation phenomenon through π-stacking and hydrogen bonding interactions, asphaltene aggregates can form a thin layer at the crude oil-brine interface through noncovalent interactions such as -O-H···O hydrogen bonds and/or alter the wettability state of the solid surface from initially water-wet into mixed-oil wetting. Here, we probe the impact of water with variety of salinities and ion types on formation of water in oil micro-emulsions, asphaltene deposition, and induced water wettability transition at micro scale. For the first time we investigate the influence of water in oil micro-emulsions on asphaltene aggregation and deposition phenomena at elevated pressure and temperature conditions. We also monitor the micro-wettability alterations of gold surface of the QCM owing to ion valency/concentration changes using state of the art ESEM imaging facility. Our results depict that owing to the substitution of divalent cations with monovalent ones, asphaltene deposition is repelled and the solid surface becomes more hydrophilic, proposing a generalizable strategy to control wettability and an elucidation for the profitability of so-called low salinity water flooding, an enhanced oil recovery methodology. For the biological applications, this study provides insights into the potential roles of ions and hydrogen bonds in the protein deposition in tissues and self-assembly interactions and efficiency of drugs against protein aggregation drivers.

## Introduction

The liquid–liquid and solid–liquid interfaces play critical roles in different physical and chemical phenomena, from lubrication and colloidal stabilisation to remediation of environmental contamination and enhanced oil recovery (EOR)^1–6^. Particularly, the water-oil and oil-solid interfaces are becoming increasingly illustrated within the scientific community.

Growth in oil and energy demand and ageing formations are some sakes why operators have been seeking various techniques for increment of oil recovery rates which are called as enhanced oil recovery methods including gas injection, water flooding, and water alternating gas (WAG) injection scenarios^7–9^. Furthermore, it has been explored that the oil production yield could be increased with decreasing the ionic strength of the flooded brine, i.e. without using costly and possibly detrimental chemistries, which is named low salinity water flooding (LSWF)^10–13^. Still, the microscopic drivers of the oil recovery enhancement owing to LSWF are under intense debate. Competitive wetting of oil and water on solid surfaces and respective wettability transitions are advocated as keys to increase recovery during water injection process. A solid is usually referred to as thoroughly water-wet (hydrophilic), oil-wet (lipophilic), or mixed wet. Several researchers also investigated crude oil–water interfacial rheology regarding the stability of water-in-oil (W/O) microemulsions as one of the drivers of water flooding induced recovery increment^14,15^. These researchers inferred that the elasticity of the liquid–liquid interface to stability of microemulsions is owing to formation of interfacial thin layers from surface active species in the oil respect to the ion concentrations in the aqueous phase. These surface-active polar species, which play important roles in wettability alterations and formation of W/O microemulsions, are asphaltenes in crude oil.

Polycyclic aromatic hydrocarbons (PAHs), which are ubiquitous organic matters constructed by some fused carbon aromatic rings and hydrogen atoms^16,17^. PAHs are regularly utilised in a vast range of industrial applications like lubricant and chemical industries, pharmaceutics, amidst a multitude of others^18–20^. Asphaltenes are PAHs which have mighty sheet-like structures of interlocked heterocyclic aromatic rings and contain both polar and nonpolar species. Additionally, they constituted by 11 ± 4% heteroatoms like O atoms (exist in carboxylic acid, hydroxyl, carbonyl groups, phenol, etc.), S atoms (in sulfoxide, thiophene), and N atoms (in pyrrole, pyridine) along with trace amount of coordinated heavy metals such as V, Ni, Fe. The surface active functional groups like carboxylic acids (–COOH) in asphaltenes are well-known to be adsorbed at the oil–water and oil–solid interfaces and can construct organized thin film sections^21–23^. Asphaltenes tend to precipitate out of the solution (2–10 nm), aggregate (>10 nm), and deposit onto the solid surfaces during the production/transportation of crude oil owing to the alterations in equilibrium conditions, e.g., composition, temperature, and pressure.

During water/gas injection scenarios, asphaltene may accumulate at the liquid–liquid and solid–liquid interfaces and form microemulsions (due to interfacial tension (IFT) reduction)^24^ and alter the wettability of solid surfaces, respectively. Yet, the mechanisms of these two phenomena through asphaltene polar species are not completely understood because of the complexity of asphaltenes molecular structures and their behaviour under realistic conditions. Some studies were performed to investigate the influence of asphaltene nanoaggregates on viscoelastic characteristics of the oil–water interface as well as the influences of some test conditions including the temperature and pH of the aqueous phase on the rate of asphaltene film formation at the interface^25–27^. Moradi and Alvarado^26^ studied the influence of the temperature on the kinetics of asphaltene film formation. They inferred that the asphaltene particles move towards oil–water interface up to 2 times quicker when the temperature is increased from 25 °C to 50 °C at which the diffusion is one of the dominating drivers. Kazemzadeh *et al*.^27^ investigated the effects of various test conditions such as pressure and water chemistry on emulsification. They observed that when the oleic phase, with more acidic matters rather than basic ones, is in contact with aqueous phase at acidic pH and high H^+^ environment, the polarity of asphaltene nanoaggregates can be raised which leads to further movement of asphaltenes towards the oil–water interface.

Crude oil is co-produced with water during the water flooding processes which contains dissolved salts of sodium, potassium, calcium, and magnesium, particularly in the case of LSWF. In this regard, systematic investigation of asphaltene-brine interactions is of interest to build fundamental understanding of the role of brine in asphaltene deposition. Such indispensable investigations are still in their early stages. In the past decade, a limited number of controversial research studies have been undertaken to understand the effect of water emulsions on asphaltene precipitation and deposition. From a review of available literature^28–31^, it appears that opinion differs significantly as to which experimental methods should be utilised to determine the asphaltene stability and deposition tendency, which conditions should be applied and how data should be interpreted. Several workers have claimed that the existence of water microemulsions had no remarkable influence on the asphaltene precipitation^28,31^, while others concluded that water increases the solubility of the asphaltenes in the system and delays the asphaltene aggregation and deposition phenomena^29,30^. All the aforementioned research studies have been conducted with the use of deionized water in the absence of any salts. They also used n-alkanes as precipitants which could not emulate the real field conditions. This makes the outcome of the investigation not appropriately precise, since our own recent work proved that asphaltene molecular structure can be varied based on the operating conditions which is a key to asphaltene behaviour at liquid-liquid and solid-liquid interfaces^32^. There are no reports of the effect of brine with varying ionic strengths on asphaltene aggregation and deposition phenomena at elevated pressure and temperature systems. No authentic model material has been developed that can imitate asphaltene behaviour, and this commands the use of genuine samples in any scientific illustration. Herein, we utilised real oil sample and asphaltenes obtained from that parent sample during the experiments. Understanding the behaviour of asphaltenes at interfaces lies on the interactions between asphaltene nanoaggregates/brine in original crude oil. Therefore, it is vital to meticulously investigate these interactions under different salinity conditions which has remained a gap in the literature. The aim of the present work is to study the interactions between water and asphaltene molecules in presence of different ions using newly measured high-pressure high-temperature quartz crystal microbalance (HPHT-QCM) data along with various analytical techniques with particular focus on low salinity water/gas injection for increment of oil production. This work documents an extensive set of HPHT-QCM experiments to inquire the impact of brine with variety of ionic strengths on asphaltene aggregate size, deposition and micro scale wettability transition of QCM surface. Furthermore, our study sheds light on the mechanisms underpinning LSWF and elucidates how large a role water molecule acts at fluid-solid interface.

## Results and Discussion

### Characterisation of asphaltene and the parent oil

Material properties of the crude oil BR including its density *ρ*_*o*_, its viscosity *µ*_o_, asphaltene content *f*, asphaltene density *ρ*_*a*_, and water content (ppm) as well as its saturates, aromatics, and resins contents are provided in Table 1. The probability of asphaltene precipitation phenomenon can be estimated using Colloidal Instability Index (CII) based on saturates, aromatic, resins and asphaltenes (SARA) contents which is expressed as CII = (saturates + asphaltenes)/(resins + aromatics). The asphaltene nanoaggregates are assumed to be stable in the solution for CII < 0.7, while they are unstable mainly for CII > 0.9^33^. For the studied crude oil BR, the asphaltene particles are unstable in terms of phase equilibrium^34^ and can start precipitation and aggregation phenomena, since its CII is 0.924. The elementary analysis data for the asphaltene fraction in w/w % are shown in Table 2.

**Table 1:** Material properties and SARA analysis of the petroleum fluid, BR: oil density *ρ*_*o*_, viscosity *µ*_o_, asphaltene content *f*, asphaltene density *ρ*_*a*_, water content (ppm).

Petroleum Fluid	*ρ*_*o*_ (g.mL^−1^)	*µ*_o_ (cP)	*ρ*_*a*_ (g.mL^−1^)	water content (ppm)	*f* (g.g−1) (n-C7 induced)	Saturates (wt%)	Aromatics (wt%)	Resins (wt%)
BR	0.828	12.15	1.05 ± 0.07	845	0.0312	44.91	37.62	14.35

**Table 2:** Elemental contents of the asphaltenes isolated from petroleum fluid BR (w/w%).

Asphaltenes	C	H	S	O	N_H_/N_C_^*^
BR	84.38	7.79	2.96	4.87	1.03

*H/C is the atomic ratio of hydrogen and carbon.

**Figure 1:**
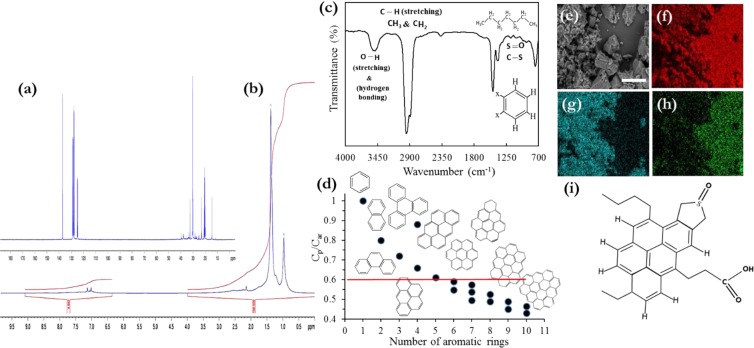
Characterisation of asphaltene isolated from crude oil BR, (**a**) representative ^13^C 100.6 MHz NMR spectra, (**b**) representative ^1^H 400.1 MHz NMR spectra, (**c**) FTIR spectra of asphaltene with representative structures assigned to related spectra range, (**d**) Determined C_p_/C_ar_ ratio as a function of total number of aromatic rings in studied asphaltene molecular structure, (**e**) ESEM micrograph of asphaltene with white scale bar of 20 µm, EDX elemental mapping of (**f**) C *k* mapping, (**g**) S *k* mapping, and (**h**) O *k* mapping, (**i**) hypothetical asphaltene molecular structure derived based on the attained advanced spectroscopy data. No effort was accomplished to fit the structure to the molecular weight, solubility, or further physical circumscriptions.

Figure 1e–h show the ESEM/EDX analysis results of the asphaltenes. Figure 1e shows agglomeration of irregular shape asphaltene particles (with an average length of ~3.7 μm) owing to high aromaticity in presence of smooth surface asphaltenes (large-sized particles of ∼12.4μm). Qualitative analysis of the asphaltene composition and its elemental mappings are given in Fig. 1f–h. Both S and O are the constructor heteroatoms and evenly distributed throughout the asphaltene deposits.

FTIR spectra of the asphaltene moiety was measured and indicated in Fig. 1c. Three characteristic peaks at 1670, 1417, and 1372 cm^−1^ were caused by the C=C stretching, the stretching vibration of aromatic rings, and CH_3_/CH_2_ deformation in the asphaltene. The characteristic peaks at 3490, 1760, and 1027–130 cm^−1^ were attributed to three oxygen and sulfur-containing groups on the asphaltenes of –O–H, –C=O of carboxylic acid, –S=O/–C–S and –C–O of sulfoxide and secondary alcohols/ethers, respectively. Three adjacent hydrogens at 760 cm^−1^ seemed to be agglutinated into the 4 H peak at 745 cm^−1^. There is a small peak ascribed to the long alkyl chains at 700–720 cm^−1^, which denotes that the asphaltene molecule has trace amount of alkyl chains attached to its structure. The presence of a C_aro_ − CH_3_ attached to the condensed asphaltene aromatic structure can be observed at ∼2730 cm^−1^. The related bonding in the asphaltene molecular structure are also depicted in Fig. 1c. The liaising major vibrational assignments are presented in Table S1 (Supporting Information). For further detailed structural characterisation, ^13^C and ^1^H NMR spectra are utilised and presented in Fig. 1a,b, respectively. The chemical shifts δ (in ppm) for ^1^H NMR corresponding to the characteristic signal intensities are 0.30–0.95 (γ-CH_3_ hydrogens to aromatic rings), 1.00–1.40 (β-CH_3_, β^+^-CH_3_ paraffinic hydrogens), 1.40–2.00 (β-position CH/CH_2_ to aromatic rings, naphthenic –CH/CH_2_), 2.00–2.90 and 2.90–4.50 (α-CH_3_, α-CH/CH_2_), 6.50–9.00 (aromatic hydrogens). The related chemical shifts for ^13^C NMR corresponding to the characteristic signal intensities are 0–70 ppm (aliphatic carbon, C_al_), and 90–180 ppm (aromatic carbon, C_ar_). The assignments of ^1^H and ^13^C chemical shifts in asphaltene NMR spectra discussed above are listed in Tables S2 and S3, respectively. An integration of ^1^H and ^13^C NMR spectra at various chemical shifts can succour us to determine some average structural parameters like the shape factor of asphaltene aromatic sheet, Φ (C_p_/C_ar_), which is the ratio of number of peripheral aromatic carbons C_p_ to aromatic carbons based on the procedures demonstrated by Joonaki *et al*.^32^ and others^35,36^. Figure 1d shows the aromatic structures for various C_p_/C_ar_ ratios which are acquired using fluorescence and quantum determinations^37^. The Φ value is related to the total number of rings and the condensation degree of asphaltene aromatic cores. Herein, the achieved value of Φ ≈ 0.59 reveals that the possible number of aromatic rings is 6 per sheet for one fragment asphaltene molecule. Based on all analytical findings, we can signify the most probable asphaltene molecular structure which is observed in Fig. 1i.

### Formation of water in oil micro-emulsions: the roles of asphaltenic compounds at water/oil interface, ionic strength, and ion valency

**Figure 2:**
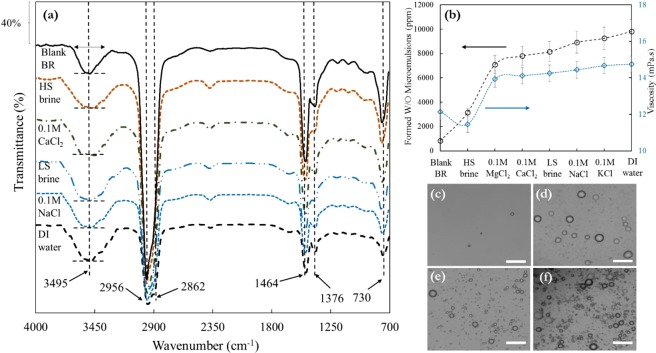
Formation of spontaneous asphaltene stabilised water in oil micro-emulsions and its effect on the fluid viscosity. (**a**) FTIR spectra of blank petroleum fluid and after contact with DI water, 1 M HS and 0.1 M LS brines, and CaCl_2_ and NaCl brines with ionic strength of 0.1 M. The broad band between 3200 and 3750 cm^−1^ assigned to hydroxyl –O–H**···**O bonding with contribution of R–COOH (**b**) Water in oil (W/O) micro-emulsions contents for blank oil and after contact with DI water and various brines with different ionic strengths and respective viscosity data. Microscopic images of the W/O micro-emulsions for (**c**) blank crude oil BR, (**d**) large 1 M HS brine droplets, (**e**) smaller 0.1 M LS brine droplets compared to HS brine, and (**f**) tiny DI water droplets. The white scale bar in microscopic images is 100 µm.

The IR Spectroscopy analysis is very significant to identify the hydrogen bonding networks in water, water-asphaltene and hydrated cations in brine solutions. This type of analysis will enable us to identify the hydrogen bonds between the –COOH/–C–S/–S=O groups of the asphaltene and the water molecules, and also to grab the alterations of O–H stretch vibration that are known for their sensitivity to the strength of the hydrogen bond network. To recognise the influences of ionic strength and ion valency on hydrogen bonding between the water and asphaltene molecules at water-oil interface, we obtained the IR vibrational spectrum for the crude oil BR in contact with various brine solutions which are shown in Fig. 2a. From the IR spectra, we denote that there is roughly one realm of 3200 and 3750 cm^−1^ (broad envelope, dominated hydroxyl –O–H**···**O bonding) which undergoes pronounced changes in their intensities owing to alterations in ionic strength of water from DI to HS brine and monovalent [Na^+^] to divalent [Ca^2+^] cations leading to multiplicity variations of H-bonding species. The intensity of the characteristic peak at ∼3495 cm^−1^ for four brines is clearly increasing owing to reducing the salinity and ion valency which result in accumulation of asphaltene nanoaggregates at water-oil interface and construction of stronger H-bonding network between carboxylic acid functional group of asphaltenes and water molecules. The lack of significant changes in intensity of aromatic C=C in presence of water droplets can explicitly impeach the recent assertions of potent asphaltene adsorption at the interface owing to the π-electron interactions between the vast PAHs and the water molecules^38^. The –COOH and/or –OH containing moieties like asphaltene molecules have attitude to be adsorbed at the interface and decrease the interfacial tension strikingly. However, the density of delicate formed film relies on their innate size and shape, moreover, on their amphiphilicity. Therefore, the interface can spontaneously bend to incorporate more asphaltene molecules. An asphaltenic-laden water-oil interface might spontaneously bend toward either the brine or the oleic phase, and this signifies the rivalry between the interaction district of the polar aromatic group and the aliphatic chains. In the existing occasion, the asphaltene molecules comprise various surface active functional groups and short hydrophobic tails (Fig. 1i) which lead to formation of W/O micro-emulsions.

As can be seen from Fig. 2b, we found that the formation of spontaneous water in oil micro-emulsions and asphaltene nanoclusters at water-oil interface is a potent function of ionic strength of brine solutions. Reducing the salinity of the aqueous phase and utilisation of monovalent ions instead of divalent ones would result in delaying the coalescence phenomenon and augmenting the emulsions stability accordingly. The microscopic images reported in Fig. 2c–f illustrate that the spontaneous emulsification phenomenon was enhanced as the concentration of salts and divalent ions decreased and presence of these water micro-droplets in oil might affect the asphaltene aggregation and deposition drivers which will be discussed later herein. We daresay that the variations in droplet size and concentration of water droplets for the LS and HS brine, solutions with ionic strength of 0.1 M [NaCl, KCl] and 0.1 M [CaCl_2_, MgCl_2_] are pertinent to the arrangement of asphaltene molecules at the water-oil interface^39^ and the ensuing interface interactions (Fig. 2b–f). We denote that Mg^2+^, Ca^2+^ with higher valence than K^+^, Na^+^ had less stable brine micro-emulsions. It is postulated that the presence of salts may promote the asphaltenic species desorption from the water-oil interface and reduce the hydration of the polar functional groups. In the case of DI water, the droplets stayed discrete, while increasing the ionic strength and valency would tend to brine droplet weight and sizes increment leading to prevail repulsive interactions and attain early onset of coalescence. The charge inversion may play a critical role on the adsorption behaviour of the asphaltenes at the fluid-fluid interface. In Fig. 2b, we show the viscosity of crude oil in contact with DI water and brine with different ionic strengths. Bulk rheology measurements ascertain that BR oil to be a Newtonian fluid with viscosity of μ = 12.15 mPa·s at the shear rate of between 10 s^−1^. Up to 3160 ppm HS brine concentration, the viscosity decreases ~6% in comparison with the sample without water augmentation. Above 4000 ppm, with reducing the salinity and valence of the cations the viscosity increased ~21% and 22% for KCl brine with ionic strength of 0.1 M and DI water, respectively. The initial decrease in the viscosity could be declarative of the presence of smaller asphaltene aggregates^40^ owing to addition of HS brine compared to blank BR oil. Then the viscosity increment might be depicted as the structural alterations caused by the abundance of water molecules.

### The effect of water with/out various ionic strengths on asphaltene aggregation

**Figure 3:**
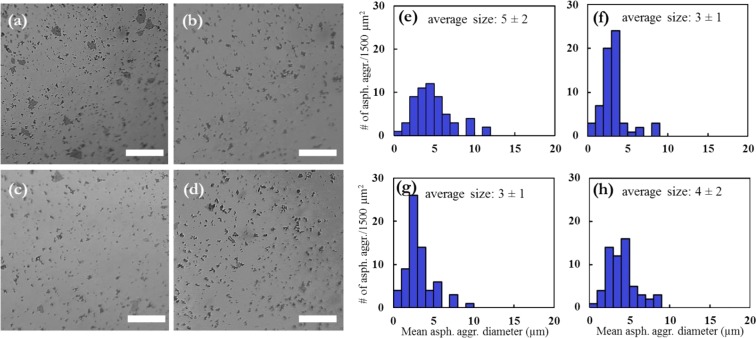
Images of asphaltene aggregates for (**a**) blank oil, and in presence of (**b**) DI water, (**c**) 0.1 M LS brine, (**d**) 1 M HS brine. Here the white scale bar in micrographs is 10 µm. The particle size distribution of asphaltene aggregates for (**e**) blank oil BR, and with (**f**) DI water, (**g**) LS brine, and (**h**) HS brine. The particle sizes are counted with ImageJ.

We determine the size of asphaltene aggregates using petrographic micrographs in order to demonstrate the influence of brine solutions with various ionic strengths. Figure 3a–d indicate microscopic imaging results for blank BR, DI water, LS, and HS brines. No significant discrepancy can be observed in the average size of asphaltene aggregates when comparing the images from the influence of ionic strength. The average size of the aggregates augmented with salinity of brine. We also determined the quantity of asphaltene aggregates and their size distribution at each image with/out brine solutions by ImageJ and depict them in Fig. 3e–h. For the sample without presence of any aqueous phase in the system, albeit the size range is between 1 and 12 μm the average size of aggregates is ~5 μm and the asphaltene aggregates are chiefly concentrated ~4 μm (Fig. 3a,e). In the presence of DI water, the size range is from 1 to 9 μm with an average of ~3 μm (Fig. 3b,f).

As can be observed from Fig. 3c,g, after addition of LS brine although the size distribution of asphaltene aggregates was different (with larger aggregates), the average aggregates size was ~3 μm the same as DI water. We also conducted the measurements in presence of HS brine and perceived some larger aggregates compared to LS and DI water with the average aggregates size of ~4 μm as shown in Fig. 3d,h. The literature illustrated the impact of water on the asphaltene particles size in solvents, particularly in toluene^41,42^. The presented micrographs, for the first time, attain new data on the influence of brines with various ionic strengths on the size of asphaltene aggregates. Asphaltenes can form a networking soft structure at liquid-liquid interface. This deformable structure could not be packed and tight owing to the steric hindrance between asphaltene aggregates. The electrostatic interactions at the fluid-fluid interface can elucidate the impact of low salinity brine on the viscoelasticity and asphaltene structure of the interface. Therefore, the electrical double-layer expansion^43,44^ might be the symptom for vast content of crude oil surface active species (e.g. asphaltenes, resins) at the liquid–Liquid interface. A diffuse ionic stratum can be constructed due to adsorption of the ions to the electrically negatively charged water-oil interface which can spotlight all the interactions with the bulk solution. The Debye length, κ^−1^, which the stoutness of the effectual layer is pertinent to, defined as:

1 $${k}^{-1}=\sqrt{\frac{{K}_{B}T{{\epsilon }}_{r}{{\epsilon }}_{0}}{2{N}_{A}{e}^{2}I}}$$

where *k*_B_ is the Boltzmann constant*, T* is the temperature, *ϵ*_r_ the dielectric constant of brine, *ϵ*_0_ the permittivity of free space, *N*_A_ the Avogadro number, *e* the electron charge, and *I* the ionic strength (eq. 10). The κ^−1^ is reversely related to the brine ionic strength. The polar surface-active moieties like asphaltenes adsorb onto the water-oil interface without ions owing to their polar functional groups formed hydrogen bonding network at the interface (as depicted in Fig. 2a) which can cause prevention of competing self-assembly contacts and aggregates growth. The diffuse stratum is flattened at low ionic strength leading to the potent screening and the asphaltenes are adsorbed and organized at the fluid-fluid interface by electrostatic interactions. The κ^−1^ is tenuous at high ionic strength; the opposing ions decrease the charge screening and also disturb the hydrogen bonding structure at periphery of –COOH/–OH containing groups at the oil-water interface which results in reduction of asphaltene-water interactions at the interface and facilitation of multiple asphaltene intermolecular π-stacking/H-bonding interactions and particle size increment accordingly.

### The role of brine solutions with different salinities on asphaltene deposition and respective micro-scale wettability transition of solid surface

**Figure 4:**
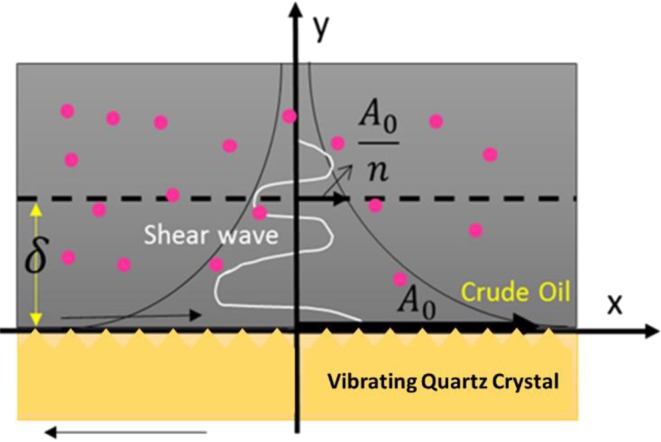
Amplitude and acceleration decay at the quartz crystal-oil interface.

Herein we provide the results from extensive sets of HPHT-QCM measurements to illustrate the influence of water with/out different ion concentrations on asphaltene deposition. To the best of our knowledge, there is no report of such data in the literature. The schematic of the HPHT-QCM setup is given in Fig. S1. The mass alteration of the QCM owing to the interactions respect to its surface can lead to resonance frequency (RF) changes. For liquids, the change in RF is also related to the viscosity and density of the surrounding medium, according to the following equation^45,46^:

2 $${\rm{\Delta }}f=-\,{f}_{0}^{3/2}{[\frac{{\mu }_{l}{\rho }_{l}}{\pi {\rho }_{q}{\mu }_{q}}]}^{1/2}$$

where *Δf* is the frequency change (Hz), *f*_0_ is the frequency of oscillation of unloaded crystal, *ρ*_*l*_ is the density of the liquid in contact with the electrode, µ_*l*_ is the viscosity of the liquid, *ρ*_*q*_ is density of quartz (*ρ*_*q*_ = 2.648 g.cm^−3^), and *μ*_*q*_ is shear modulus of quartz for AT-cut crystal (*μ*_*q*_ = 2.947 × 10^11^ g.cm^−1^.s^−2^). When the QCM is in contact with the crude oil, the vibration amplitude and also the acceleration decay exponentially from the crystal surface into the crude oil as shown in Fig. 4. It is mathematically illustrated by the equation below^47^:

3 $$A(y)={A}_{0}\exp (-\frac{y}{\delta })$$

where *δ* is the penetration depth which is equal to ${({\mu }_{l}/\pi {f}_{0}{\rho }_{l})}^{1/2}$ defined as the effective thickness of the crude oil that is driven to move by the vibrating crystal with a displacement decaying exponentially, and *A*_0_ is maximum vibration amplitude at the centre of the crystal.

**Figure 5:**
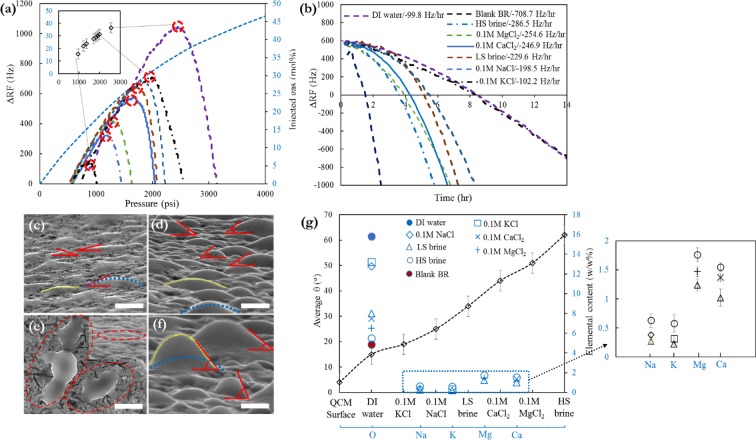
The effect of water with/out various ionic strengths and ion types on (**a**) AOP shifting and related GOR changes, and (**b**) asphaltene deposition rate onto the QCM surface. ESEM micrographs of micro-droplets of water on gold plate of QCM surface in presence of (**c**) DI water, (**d**) 0.1 M LS brine, (**e**) no water, plain surface, and (**f**) 1 M HS brine illustrating water contact angle variability owing to ionic strength changes. The white scale bar depicted in images is 20 µm. (**g**) the average contact angle values of water micro-droplets on the surface with/out presence of brines with different salinities and ion types and respective elemental analysis of trapped brine sandwiched between asphaltene deposits and the QCM surface.

Lower the vibration amplitude and acceleration are obtained owing to larger distance (y) from the QCM-oil interface. A certain small volume of crude oil placed at distance δ from the QCM-oil interface will generate a frequency alteration *n* times smaller than that generated by the same volume of crude oil placed in direct contact with the quartz crystal surface. The significant fact is that by measuring small changes in RF, tiny amount of asphaltene deposits in nano-gram range can be detected. Under the real field situation, the asphaltene deposition challenge happens due to gas injection scenarios or depressurisation process. We conducted the tests with injection of gas and oil in various gas/oil ratios (GORs) in presence of water micro-emulsions for imitating the WAG-EOR conditions and measured the asphaltene onset point (AOP) and deposition rate onto the QCM surface. In Fig. 5a, the diagram indicates ΔRF versus pressure for natural gas and BR oil at different GORs in presence of DI water with/out various salt concentrations and ion valences to monitor the AOP changes. The pressure at which the RF starts declining signifies the AOP^32,48^ which is ~954 psi at GOR of ~15.6 mol% for the blank BR crude oil. As can be seen in Fig. 5a, a distinct AOP shift is detected in presence of HS brine for which the AOP/GOR is ~1221 psia/21.8 mol%. The AOP/GOR is ~1360 psia/ 23.9 mol% for 0.1 M MgCl_2_, ~1706 psia/27.7 mol% for 0.1 M CaCl_2_, ~1820 psia/28.9 mol% for LS brine, ~1914 psia/30.1 mol% for 0.1 M NaCl, ~1990 psia/31.2 mol% for 0.1 M KCl, and ~2554 psia/36.4 mol% for DI water. Figure 5b depicts the results of the impact of brine solutions with different ionic strengths on the asphaltene deposition rate after the AOP which is RF decline versus time (ΔRF.Δt^−1^, deposition rate representative^32,48^) for BR oil with/out brine micro-emulsions at different salts concentrations. There is a drastic discrepancy in the plotted curves between the blank BR oil without any water micro-emulsion and with presence of DI water, HS and LS brines. DI water reduced the deposition rate from −708.7 to −99.8 Hz/hr. HS and LS brines decreased the rate of asphaltene deposition down to −286.5 Hz/hr and −229.6 Hz/hr, respectively.

The influence of water/brines in preventing and/or delaying the asphaltene precipitation and deposition phenomena is perceived for the BR oil; we elucidate that the influence is not particular for specific ionic strength and ion valency. Based on the attained results, plainly the delay in asphaltene precipitation and deposition rate decrement are potently relied on the concentration of salts in brine solutions. Lower ionic strengths and ion valency resulted in higher effect on preferable AOP shifting and deposition rate reduction. It is worth noting that a distinguished fluctuation is noted at high concentration of water in oil micro-emulsions (DI water) possibly owing to structural alterations caused by water molecules in asphaltene nanoaggregates. There are two conceivable elucidations for this dramatic influence of water/brines on QCM asphaltene experiments. The first hypothesis is that the water molecules interact with asphaltene molecules, curb further intermolecular interactions and reduce the size of asphaltene aggregates which has been demonstrated in previous section. The second possible driver is that the water/brines construct a thin film layer at periphery of the QCM surface and prevent asphaltenes from depositing. In order to elucidate if the second driver is dominant, we monitored the micro-wettability alterations of gold surface of the QCM due to ion valency/concentration changes. Furthermore, it is crucial to note that during the experiments the pH value was kept at 6 at which the –COOH moieties of asphaltenes can be activated through dissociation phenomenon^49^. Due to deprotonation of the most –COOH functional groups at that pH, they are negatively charged and can interact potently with Ca^2+^ and Mg^2+^ divalent cations. Therefore, cationic complexes with carboxylates can be formed, and it is plausible that this chemical binding between carboxylate anions of asphaltenes and cations joined to another molecule also leads to aggravation of asphaltene precipitation and aggregation phenomena and greater amount of asphaltene deposits onto the QCM surface through ion bridging process (Fig. 5a,b).

Figure 5c–g present the images of water droplets onto the solid surface in presence of various ions and corresponding contact angle values. In order to analyse the competitive wetting of organic species and water on the QCM surface, we measured the contact angle of water micro-droplets. The microscopic contact angles of droplets are depicted in mainly side view micrographs (Fig. 5c–f). We measured different contact angles under each condition in various regions of the solid surface to accurately determine the influence of brine salinity and ion valency on the wettability transition of the surface. Then average values of these local contact angles at micro-scale were determined which denoted as θ_*av*_ and represent the general wettability of the solid surface. To the best of our knowledge, this is the first report of such a data in the literature. Figure 5c–g illustrate the impacts of the concentrations of the combined mono and divalent cations on the micro-droplets contact angle changes. If the DI water serves as a reference with θ_*av*_ of ≈15°, the θ_*av*_ increased with increasing salinity of the water. The θ_*av*_ in presence of LS brine showed an increment from 15° to ≈34°. The only remarkable district was noticed between LS and HS brines that is ≈28°, from 34° to ≈62°. The representative extracted water micro-droplet dimensions in average for LS and HS brines are 15.8 μm (height) × 51.6 μm (width) and 20.4 μm (height) × 46.7 μm (width), respectively. We also demonstrated the effects of the valence of the cations in the brine solutions on the contact angle transitions. Increasing the concentration of [K^+^] from 0 to 0.1 M was resulting in θ_*av*_ increment from 15° to ≈19°. Due to replacement of [K^+^] with [Na^+^] at same concentration, the θ_*av*_ increased up to ≈25° which reveals the importance of cation type in addition to ionic strength. However, when [Ca^2+^] is added instead of [Na^+^], the θ_*av*_ raised to ≈44°, and 33 mM [Mg^2+^] leads to a θ_*av*_ of 51°. This evidently underlines how significant the roles of ion type and valency are in wettability alteration phenomenon. To unravel the mechanisms behind this observation, we require to consider the interactions between the cations and the charged QCM surface.

Figure 5g indicates the elemental analysis of the asphaltene deposits onto the surface and related concentrations (w/w %) of [O], [Na^+^], [K^+^], [Ca^2+^], and [Mg^2+^] to reveal the effects of water with/out various salts on the deposition rate reduction and micro-wettability changes. The [O] content increased with reducing the salinity of the brine and substitution of divalent cations with monovalent ones. It elucidates that more water molecules trapped in asphaltene deposits on the solid surface in presence of monovalent cations containing water and lower ionic strength brine. The concentrations of divalent cations are more than monovalent cations due to their sizes and hydration states leading to high strength ion-surface interactions and more potently cation adsorption onto the gold surface. The bare ion radii and hydrated ion radii for different cations utilised in this study have been presented in Table S4 (supporting information). The activity coefficients for the cations in the chloride solutions are determined using the Debye-Huckel equation^50^:

4 $$log{\gamma }_{i}=-\,\frac{A{z}_{i}^{2}\sqrt{I}}{1+B{a}_{i}\sqrt{I}}+{b}_{i}I$$

where *A* and *B* are the temperature dependant coefficients, *I* is ionic strength (eq. 10) and *a*_*i*_ and *b*_*i*_ are individual cation activity coefficient parameters which are given in Table S4. The size of the divalent cations and respective number of water molecules surrounding the cations are greater compared to the monovalent cations. The hydration happens readily for metal cations at the QCM-fluid interfaces. Water molecules can befit a substantial portion of coordination for the metal cations. The binding energy, *E*_*bw*_, of the water molecules to the divalent cations in vacuum is defined as^51^:

5 $$\,{E}_{bw}=E(cation+6water)-E(Cation)-6E(water)$$

This expression illustrates that the more negative the binding energy, the more potently the water molecules append to the cation. The free energy of hydration and the binding energy for first hydration shell (6 water molecules) are −1821 and −1395 kJ.mol^−1^ for [Mg^2+^], while they are −1500 and −1063 kJ.mol^−1^ for [Ca^2+^] cation^52^. Based on the obtained results (Fig. 5g), it can be inferred that the divalent cations adsorbed more strongly onto the surface owing to their higher concentrations in deposits and ruptured the ordered structure of water molecules and their H-bonding network at the solid-liquid interface that lead to direct molecular contact of asphaltenes and providing of free surface active sites accessible to readily interact with asphaltene nanoaggregates. This proposition is consistent with the higher concentrations of trapped divalent cations compared to monovalent cations. On the other hand, the hydration number of metal cations reduced with decrease in ion valency, and in presence of monovalent cations, more water molecules from the hydrated cations are released which increases the amount of free water molecules into the oil-water interface and oleic phase that results in further mobility of the cations. The contact angle for three phases (QCM surface-oil-water) system can be deduced from the Young’s equation^53^:

6 $${\rm{\Delta }}(cos\theta )=\frac{({\rm{\Delta }}{\gamma }_{os}-{\rm{\Delta }}{\gamma }_{ws})}{{\rm{\Delta }}{\gamma }_{ow}}$$

and,

$$({\rm{\Delta }}{\gamma }_{os}-{\rm{\Delta }}{\gamma }_{ws})\propto {\rm{\Delta }}{\rm{\Delta }}{E}_{ads}(asphaltene,\,mono\,{\rm{v}}\,di-valent\,cation)$$

where *γ*_*ow*_, *γ*_*os*_, and *γ*_*ws*_ are interfacial tensions between oil and water, oil-solid, and water-solid surfaces, respectively. The ${\rm{\Delta }}{\rm{\Delta }}{E}_{ads}(asphaltene,\,mono\,{\rm{v}}\,di-valent\,cation)$ is the alteration of the discrepancy in adsorption energy for the asphaltene molecule, relative to water molecule. It can elucidate how the surface tendency for asphaltene molecules compared to its tendency for water molecules in presence of mono/di valent cations. If ${\rm{\Delta }}{\rm{\Delta }}{E}_{ads}(asphaltene,\,mono\,{\rm{v}}\,di-valent\,cation)$ is negative the preference of asphaltene to be adsorbed onto the solid surface is higher than water molecules in presence of specific cation. Herein, for the studied case of solid-liquid-vapour phase using ESEM micrographs, the Eq. (6) can be expressed in terms of work of adhesion:

7 $${W}_{SL}={\gamma }_{LV}(1+cos\theta )$$

where *W*_*SL*_ = *γ*_*LV*_ + *γ*_*SV*_ · *γ*_*SL*_, and S, L, and V stand for solid, liquid, and vapour phases. The Eq. 7 can be rewritten in terms of binding energy per unit area as follows:

8 $$1+cos\theta =\frac{{E}_{b-asph}}{A{\gamma }_{LV}}$$

where A is surface area, and ${E}_{b-asph}$ is the binding energy of the asphaltene molecules to the QCM surface and can be defined as:

9 $${E}_{b-asph}={E}_{surf}+{E}_{asph}-{E}_{surf.asph}$$

where ${E}_{surf.asph}$ is the total energy of the adsorbed asphaltene molecule on the gold surface of the QCM, ${E}_{surf}$ is the total energy of the gold surface alone and ${E}_{asph}$ is the total energy of the asphaltene molecule in a vacuum. Taking into account ${E}_{b-asph}$ and ${\rm{\Delta }}{\rm{\Delta }}{E}_{ads}(asphaltene,\,mono\,{\rm{v}}\,di-valent\,cation)$, previously discussed preference of cations for adsorption, and achieved contact angles presented in Fig. 5g could explain why monovalent cations are more effectual in suppressing the asphaltene adsorption on the gold surface and reducing the corresponding contact angles of water micro-droplets. Through similar drivers, Ca^2+^ and K^+^ are more efficient in inhibiting the adsorption of asphaltene nanoaggregates onto the QCM surface compared to Mg^2+^ and Na^+^, respectively. Generally, the binding affinity of alkali and earth alkaline cations in our thin film systems both at solid-liquid and liquid-liquid interfaces is presumably to be a plural influence pertinent to the ion valency, the hydration of the surfaces and ions and the interacting surfaces charges. Our results concur with some reports in the literature on the effect of cations on adsorption of proteins^54^ and stearic acid^55^ onto the mica surface.

**Figure 6:**
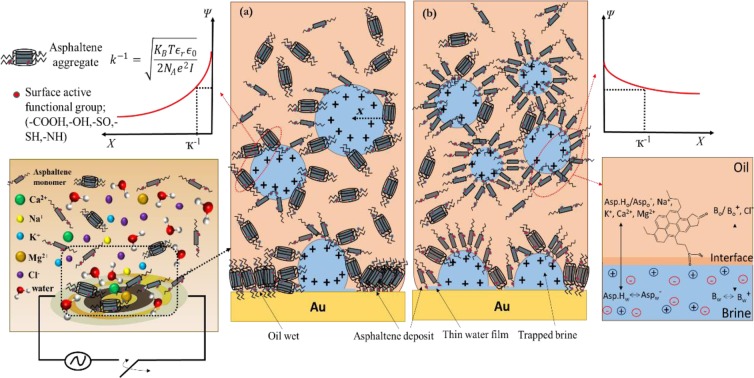
Model representation of the molecular scale phenomena that elucidate the effect of ionic strength on asphaltene aggregation, deposition, formation and stability of W/O micro-emulsions, asphaltene interactions at water-oil interface and microscopic wettability transition for (**a**) high ionic strength, and (**b**) low ionic strength brines with respective Debye length (κ^−1^). Ions can alter the electrostatic potential (*ψ*). Micro-brine droplets and thin water film sandwiched between oil-asphaltene deposits and the QCM solid surface, owing to cations (mainly multivalent ones) induced rupture of protective water films the asphaltene aggregates are allowed to adhere and trapped patches of brine are left on the surface. The ions water shielding and anions (in (a) & (b)) are not depicted for the sake of lucidity.

Figure 6 illustrates all the proposed derived mechanisms of asphaltene deposition in presence of brines with variety of ionic strengths, formation of water in oil micro-emulsions, and micro-wettability transitions all observed in this study. We caution that our results require further investigations down to molecular scales and have set the stage for density functional theory (DFT) calculations and molecular dynamic simulations with consideration of all the equations (5)–(10) to build a better understanding of the proposed mechanisms which is currently under investigation from the current authors.

The impacts of lower ionic strengths on the oil displacement and adsorption phenomena were identified as a critical factor for improved oil recovery during water flooding into the oil reservoirs. Different parameters like fluid-fluid and solid-fluid interactions would affect the yield of low salinity water injection process. Wettability transition and formation of water in oil emulsions (IFT reduction) are the most significant drivers of low salinity enhanced oil recovery methodology. Effectual improved oil recovery during low salinity waterflooding requires a fundamental understanding of the mechanisms behind the oil surface active species adsorption onto the water-oil and solid-oil/water interfaces in presence of various ion types and ion valences. This is the first study to illustrate the effect of water in oil micro-emulsions in presence of different ionic strengths and dissolved chloride salts of a variety of alkali and earth alkaline cations on asphaltene aggregation and deposition phenomena under realistic conditions. It also opens a door to a better understanding of the key low salinity EOR drivers. Additionally, the results demonstrate the first experimental evidence backed by analytical results on the role of ion valency on micro-wettability transition in presence of asphaltenes. Although the thickness of the Debye layer κ^−1^ (eq. 1) increases with decreasing the ionic strength, it stays in a domain where the prevailing forces are controlled by stunted range of interactions that are not thoroughly captured by Derjaguin–Landau–Verwey–Overbeek (DLVO)^56^ theory. Without requirement to invocate any model, our present study clearly implies that the ion type in addition to ion concentration has drastic influences on wettability and asphaltene interactions at liquid-liquid and solid-liquid interfaces.

Our results have potential applications in other systems as well. Mechanisms involving interface interactions are of significant relevance for the understanding and control of biological systems. We have acquired no debates against the mechanisms being expandable to the biological macromolecules interactions. The conformation of tiny and mighty organic species, proteins as biological macromolecules, as well as the specificity of molecular recognition are governed by the identical types of intermolecular forces including attractive and repulsive forces as asphaltenes have. Therefore, it could be conceivable to elucidate the three-dimensional (3D) structures of these species and their recognition specificities based on the mutual principles. The hydrogen bonds play a pivotal role in every field of biochemistry^57,58^. The reiterated occurrence of interactions between aliphatic chains, π-stacking and H-bonding in biological macromolecules such as proteins^59,60^ alludes a functional role for them in describing the structures stability, in vast molecular recognition events as well as the folding drivers of macromolecules. This denotes that the same interactions as asphaltenes are presumably to happen on the cell surface. In summary, our research study outlines testable mechanisms for protein adsorption, protein-protein and protein-water interactions, and protein aggregation inhibition through progress of nanomedicine design. Further work on this topic through quantum mechanical simulations will address confirmation of hypothesis and propositions throughout this study with hitherto unaccessed molecular level resolution.

## Materials and Methods

We utilised a petroleum fluid, that we call BR, and determined its asphaltene content as well as its molecular weight, density and viscosity. The QCM experiments were conducted using AT-cut (optimized for 90 °C) 5 MHz quartz crystal coated with gold purchased from Testbourne Ltd. The diameter of the crystal is 25.4 mm, while the front electrode diameter is 12.7 mm, the crystal thickness is 333 µm, and the crystal surface roughness is 50 Å. The sensors were cleaned using anhydrous heptane and toluene (>99%) from Sigma-Aldrich (used as received) followed by rinsing with deionised water and blow-drying with nitrogen to remove all surface-active impurities. To be more realistic for mechanistic studies we precipitated asphaltenes by adding natural gas in our QCM test set-up. The utilised natural gas composition is as follows (*Mole %*): N_2_ (*1.84%*), C_1_ (*89.94%*), CO_2_ (*0.91%*), C_2_ (*5.32%*), C_3_ (*1.45%*), iC_4_ (*0.20%*), nC_4_ (*0.21%*), iC_5_ (*0.07%*), (nC_5_) + C_6_^+^ (*0.06%*). After the deposition test, the gas induced asphaltene deposit from the QCM surface was extracted and dried for characterisation. The detailed procedure has been presented in our previous articles^32,48^. The asphaltene content of BR is determined by adding HPLC-grade anhydrous *n-*heptane (>99%, Sigma Aldrich) and define *X* (mL.g^−1^) as the *n-*heptane ratio, showing the volume of *n-*heptane added to 1gr of petroleum fluid. After mixing of 4gr crude oil with 160 mL *n-*heptane, *X* = 40 mL.g^−1^, for 10 min in a sonicator bath and allowed to be equilibrated for 24 hr before separation of asphaltene fraction, the solution was filtered through a 0.2 mm pore-size cellulose nitrate whatman filter followed by washing with excess *n-*heptane until shiny black asphaltenes were appeared. The filtrate is then collected, dried and weighed to give the n-heptane asphaltene content as presented in Table 1. The density of the crude oil BR*, ρ*_*o*_, is measured using a densitometer (Anton Paar). The density of the asphaltene, *ρ*_*a*_, is determined by preparation of a solution of 0.05 g asphaltene in 20 mL toluene and measuring the density of the solution. The viscosity measurements are conducted using a stress controlled rotational-type rheometer (Anton Paar, Physica MCR 301) with the aid of 25 mm diameter and 1° angle cone-plate geometry (Cones CP50–1). The water content results in ppm, are measured by a coulometer (Karl Fischer 331 coulometer). Deionized (DI) water was produced using an ELGA DV 25 Integral Water Purification System. For all the brine phases, we utilised freshly DI water in which different combinations of the following salts were dissolved: NaCl, KCl, CaCl_2_, and MgCl_2_, which were purchased from Sigma-Aldrich and used as received (Analytical grade, purity > 99.5%). Single and multi-salts solutions are within the range of 100 mM to 1 M ionic strengths to mimic low salinity water and sea water which are injected into the reservoirs for secondary/tertiary oil recovery. The ionic strength, defined as

10 $$I=\frac{1}{2}{\sum }_{i=1}^{n}{z}_{i}^{2}{c}_{i}$$

where *c*_*i*_, *z*_*i*_, and *n* are the concentration, the valence of the i*th* ionic species, and the number of different species, respectively. All the solutions were adjusted to pH 6 with HCl/NaHCO_3_ and NaOH (purchased from Sigma Aldrich). The amount of acid or base augmented was meagre respect to the ionic strength of the solutions. The crude oil BR was placed on top of the salt solutions thick film to increase the oil/brine contact surface area followed by inverting andante to facilitate molecular interactions at oleic-aqueous interface and obtain suitable mixing but to avoid emulsification. Some water remained at the bottom of the vials and oils were intently separated for the pursuant tests. An FEI Quanta 650 FEG SEM, with a backscattered electron (BSE) imaging detector, equipped with an Oxford Instruments X-MaxN150 mm energy dispersive X-ray (EDX) detector^32^, was used in this work. For both imaging and elemental analysis, the microscope was operated in low-vacuum mode (0.83 Torr) at 20 kV, spot size of 4.5, dwell of 10μs, and a working distance of 10 mm. Micro wettability alterations were monitored using an XL30 Environmental Scanning Electron Microscope (ESEM), fitted with a Peltier cooling stage. Since the samples were flat, with minimal surface topography, they were mounted onto a modified cooling stage (clamp) stub, thus contact angles could be observed in side view and therefore directly measured. The Peltier cooling stage was set to 5 °C, and the chamber set to pump to full ESEM wet-mode with a target vacuum of 4 Torr. In addition, the chamber was programmed to pass through five cycles of 4 to 10 Torr, to ensure that sufficient water vapour was present within the chamber to provide the wetting medium and maximise image quality. Once the chamber had reached the requisite 4 Torr, an initial image was obtained (at an operating voltage of 20 kV), and image quality improved by shortening the working distance. Once correctly set up, the vacuum was slowly decreased to 6.5 Torr, while maintaining a temperature of 5 °C. At 6.5 Torr and 5 °C, relative humidity reaches 100%, and water droplets can condense from the water vapour atmosphere within the ESEM chamber on to the surface of the sample. No direct control exists to predict where water droplets will start to nucleate, so it was necessary to observe as wide a field of view as possible, and once water droplets started to form images could be digitally recorded at higher magnifications. At this stage, care needs to be taken not to over magnify the droplets, as localised beam heating will vaporize or otherwise alter the contact angle of the droplets.

The FTIR spectra were recorded using an FTIR-4000 Series (JASCO Edition) spectrometer including a Peltier stabilized DLaTGS detector and a high output ceramic source coupled with an attenuated total reflectance (ATR) mode^32^ with high through put monolithic diamond and ZnSe. The spectral domain is 650–4000 cm^−1^with a resolution of 0.7 cm^−1^. The proton ^1^H and carbon ^13^C NMR spectroscopic analyses were conducted using a Bruker AVI400 spectrometer operating at 400.1 and 100.6 MHz for proton and carbon^32^, respectively. Toluene-d8 (99.96atom % D) was utilised as received from Sigma-Aldrich as a solvent for the NMR experiments. The proton data were obtained employing a 3.96 s acquisition time, 8278 Hz sweep width, and 1.0 s relaxation time. The carbon spectra were achieved with a 1.30 s acquisition time, a 25 125 Hz sweep width, and 2.0 s relaxation time. The carbon spectra resulted from 1024 scans. Herein the given chemical shifts (δ) are reported respect to tetramethylsilane (TMS) utilised as internal standard^32^. All stable microemulsions and asphaltene aggregates were imaged using state of art petrographic microscope using a 50 × objective with 0.25μm resolution, and asphaltene aggregate sizes were counted by ImageJ to analyse the number of aggregates of various sizes per area of 1500 μm^2^.

## Supplementary information

Water versus Asphaltenes; Liquid−Liquid and Solid−Liquid Molecular Interactions Unravel the Mechanisms behind an Improved Oil Recovery Methodology
