# Supplementary material for: Water versus Asphaltenes; Liquid–Liquid and Solid–Liquid Molecular Interactions Unravel the Mechanisms behind an Improved Oil Recovery Methodology
Source: Sci Rep. 2019 Aug 6;9:11369. doi: 10.1038/s41598-019-47782-5 (PMC6684611; doi:10.1038/s41598-019-47782-5)
Supplement: Supplementary file 1 — Water versus Asphaltenes; Liquid−Liquid and Solid−Liquid Molecular Interactions Unravel the Mechanisms behind an Improved Oil Recovery Methodology [file 41598_2019_47782_MOESM1_ESM.docx]

**SUPPORTING INFORMATION**

**Water *versus* Asphaltenes; Liquid−Liquid and Solid−Liquid Molecular Interactions Unravel the Mechanisms behind an Improved Oil Recovery Methodology**

*Edris Joonaki*^1^, Jim Buckman^2^, Rod Burgass^1^, Bahman Tohidi^1^*

^1^ Centre for Flow Assurance Research Studies (CFAR), Institute of Petroleum Engineering, School of Energy, Geoscience, Infrastructure and Society, Heriot-Watt University, Riccarton, Edinburgh, EH14 4AS, UK

^2^ Centre for Environmental Scanning Electron Microscope, Institute of Petroleum Engineering, School of Energy, Geoscience, Infrastructure and Society, Heriot-Watt University, Riccarton, Edinburgh, EH14 4AS, UK

^*^Corresponding Author E-mail: [ej5@hw.ac.uk](mailto:ej5@hw.ac.uk)

**Table of Contents**

Supplementary Figures

Fig. S1 Schematic diagram of newly designed HPHT-QCM set up ……………………………………..S3

Supplementary Tables

Table S1 Infrared spectral range assignments of the main bands observed in the asphaltene

Characterised in this study ……………………………………………………….……………………….S4

Table S2 ^1^H chemical shift correlation chart for studied asphaltene ……………………….……….S5

Table S3 ^13^C chemical shift correlation chart for studied asphaltene…………………….………….S5

Table S4 Bare ion radii, hydrated ion radii, ion size parameter å, and the individual ion

activity coefficient parameters in eq. 4 for various cations used in this study…….S6


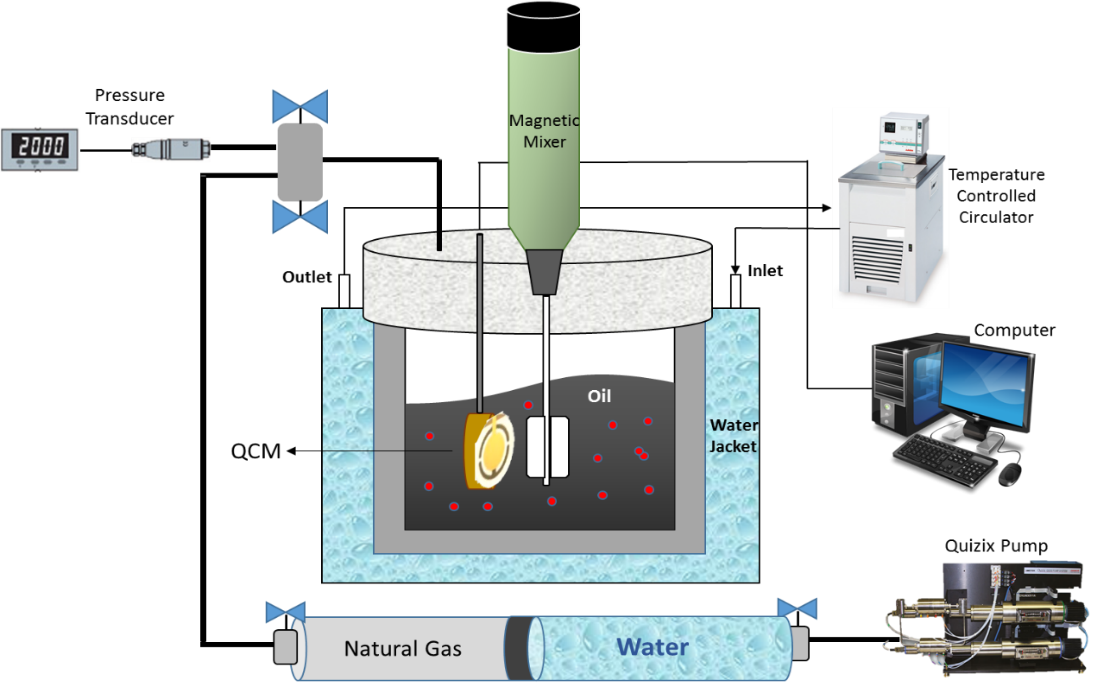


Fig. S1 Schematic diagram of newly designed HPHT-QCM set up

Table S1 Infrared spectral range assignments of the main bands observed in the asphaltene characterised in this study

| Absorption bands (cm^-1^) | Functional groups |
| --- | --- |
| 3700–3200 | Stretching vibration of the O−H bonds (the bands are broadened due to hydrogen bonding) |
| 3000–2800 | Stretching vibration of the C−H bonds in CH_3_/CH_2_ |
| 2730 | C_aro_−CH_3_ |
| 1800–1660 | Stretching of the carbonyl groups C=O bonds (COOH) |
| 1590–1620 | Stretching of the C=C bonds of aromatic moieties |
| 1370–1460 | C–H deformation in CH_3_/CH_2_ |
| ~1030 | S=O, C−S |
| 865 | C_aro_−H(1) (v) |
| 805  760  745  715 | C_aro_−H(2) (iv)  C_aro_−H(3) (iii)  C_aro_−H(3) (ii)  − (CH_2_)_n_− [n$\geq$4] |

Table S2 ^1^H chemical shift correlation chart for studied asphaltene

| Chemical shift ranges (ppm) | Integral intensity | Type of proton | assignment |
| --- | --- | --- | --- |
| 9.00 − 6.50 | vi | H_ar_ | aromatic hydrogens |
| 4.50 − 2.90 | v | H_α_(methylene/methyne) | α-CH, α-CH_2_ |
| 2.90 − 2.00 | iv | H_α_ (methyl protons) | α-CH_3_ |
| 2.00 − 1.40 | iii | H_β_ (naphthenic protons) | β-CH_2_, CH/CH_2_ naphthenic |
| 1.40 – 1.00 | ii | H_β ,_ H_β_^+^ | β-CH_3_, β^+^-CH_2_ , paraffinic CH_2_ |
| 0.95 – 0.30 | i | H_γ_ | methyl hydrogen in γ position attached to aromatic ring; methyl hydrogen in alkane |

Table S3 ^13^C chemical shift correlation chart for studied asphaltene

| Chemical shift ranges (ppm) | Integral intensity | Type of carbon | assignment |
| --- | --- | --- | --- |
| 160.0 – 137.0 | V | C_ar‑alk_ | alkyl-substituted aromatic carbon except CH_3_ |
| 137.0 – 129.0 | IV | C_ar_‑_CH3_, C_ar‑n_ | methyl-substituted aromatic carbons, carbon at junction of an aromatic and naphthenic rings |
| 136.0 – 123.0 | III | C_aa_, C_aaa_ | carbon at junction of two and three aromatic  rings |
| 130.0 – 118.0 | II | C_ar‑H_ | aromatic protonated carbons |
| 45.00 – 5.00 | I | C_al_, C_n_ | Saturated carbons, naphthenic carbons |

Table S4 Bare ion radii, hydrated ion radii, ion size parameter å, and the individual ion activity coefficient parameters in eq. 4 for various cations used in this study

| Ion | Bare Ion Radii (nm) | Hydrated Ion Radii (nm) | å/(10^-10^ m) ^[1]^ | *a* (10^-10^ m)^[2]^ | *b ^[2]^* |
| --- | --- | --- | --- | --- | --- |
| K^+^ | 0.149 | 0.331 | 3.0 | 3.5 | 0.015 |
| Na^+^ | 0.117 | 0.358 | 4.0-4.5 | 4.0 | 0.075 |
| Ca^2+^ | 0.100 | 0.412 | 6 | 5.0 | 0.165 |
| Mg^2+^ | 0.072 | 0.428 | 8 | 5.5 | 0.20 |

**References**

[1] Garrels, R. M., & Christ, C. L. *Solutions, minerals, and equilibria.* New York: Harper & Row (1965).

[2] Plummer, L. N., Jones, B. F., & Truesdell, A. H. *WATEQF: a Fortran IV version of WATEQ, a computer program for calculating chemical equilibrium of natural waters* (1976).
